# New Synthesis Routes toward Improvement of Natural Filler/Synthetic Polymer Interfacial Crosslinking

**DOI:** 10.3390/polym14030629

**Published:** 2022-02-07

**Authors:** Mahmoud M. A. Nassar, Belal J. Abu Tarboush, Khalid I. Alzebdeh, Nasr Al-Hinai, Tasneem Pervez

**Affiliations:** 1 College of Applied Professions, Palestine Polytechnic University, Wadi Alhareya, Hebron P.O. Box 198, Palestine; mnassar@ppu.edu; 2 Department of Mechanical and Industrial Engineering, Sultan Qaboos University, P.O. Box 33, Al-Khod 123, Oman; nhinai@squ.edu.om (N.A.-H.); tasneem@squ.edu.om (T.P.); 3 Department of Petroleum and Chemical Engineering, Sultan Qaboos University, P.O. Box 33, Al-Khod 123, Oman; belal@squ.edu.om

**Keywords:** bio-composites, filler–polymer compatibility, compatibilizers, functionalization, interfacial bonding

## Abstract

Among the critical issues dictating bio-composite performance is the interfacial bonding between the natural fibers and polymer matrix. In this regard, this article presents new synthesis routes comprising the treatment and functionalization of both date palm powder (DPP) filler and a polypropylene (PP) matrix to enhance filler–polymer adhesion in the newly developed bio-composites. Specifically, four bio-composite forms are considered: untreated DPP filled PP (DPP-UT/PP), treated DPP filled PP (DPP-T/PP), treated DPP filled functionalized PP using 2-isocyanatoethyl methacrylate (DPP-T/PP-*g*-IEM), and treated and functionalized DPP using 4-toluenesulfonyl chloride filled functionalized PP using 2-acrylamide ((DPP-T)-*g*-TsCl/PP-*g*-AcAm). The functional groups created on the surface of synthesized PP-*g*-IEM react with activated hydroxyl groups attached to the filler, resulting in chemical crosslinking between both components. Similarly, the reaction of TsCl with NH_2_ chemical groups residing on the mating surfaces of the filler and polymer generates an amide bond in the interface region. Fourier transform infrared spectroscopy (FTIR) is used to confirm the successful coupling between the filler and polypropylene matrix after applying the treatment and functionalization schemes. Owing to the introduced crosslinking, the DPP-T/PP-*g*-IEM bio-composite exhibits the best mechanical properties as compared to the neat polymer, unfunctionalized polymer-based bio-composite, and (DPP-T)-*g*-TsCl/PP-*g*-AcAm counterpart. The applied compatibilizers assist in reducing the water uptake of the manufactured bio-composites, increasing their durability.

## 1. Introduction

Bio-composites have undergone significant development in the last few decades. Consequently, these materials are presently being investigated for various engineering applications. Further research work committed to this topic is still needed in order to investigate the performance and other features related to bio-composites and their ingredients [1,2]. Recently, wood samples cut from a wide range of species were utilized as fillers in wood polymer–plastic composites. Because of the slow growth rate of trees and the scarcity of forest resources, alternative forms of lignocellulosic materials are being introduced to replace traditional wood flour fillers for emerging applications [3,4,5]. In general, a lignocellulosic biomass is a form of fiber or flour that is utilized as a reinforcing element in a polymer [6,7]. Natural fibers such as kenaf, flax sisal, and bamboo offer major advantages over their synthetic counterparts, such as carbon fillers and glass fillers, due to their low density, low cost, and ability to reduce mechanical wear during processing [8]. However, the degradation of natural fillers poses serious drawbacks, such as the decline of the mechanical properties over time. Nonetheless, several research studies have shown that applying a proper chemical treatment may eliminate or slow the degradation process [9,10,11].

The utilization of various types of natural fibers derived from agro-residues to reinforce or fill polymers has rapidly increased. In fact, natural fibers are considered to be viable alternatives to conventional fibers for use as reinforcements in composite materials [12]. The vast majority of manufactured bio-composites contain natural fillers in the form of fibers or particles to reinforce thermoplastic polymers, which are used for a variety of industrial products [13,14]. Commodity plastics, including polyethylene, polypropylene, and polyvinyl chloride, have gained a foothold in the bio-composite industry due to their excellent overall performance and the efficient processing methods involved, such as extrusion and injection molding [15,16]. Polypropylene (PP), a semi-crystalline thermoplastic polymeric material, is used particularly frequently because of its appealing features, which include high workability, mechanical capability, chemical resistance, and thermo-oxidative durability [17].

Among the dominant factors that hinder the wide adaptation of natural-fiber-reinforced composites is the weak filler–polymer interaction [12,18]. The polarity of lignocellulose in natural fillers is hydrophobic in nature. To overcome this problem, it is recommended to minimize the moisture content by drying the fillers prior to composite production [19,20]. Generally, the weak interfacial adhesion and limitations of the load transfer between the filler and the polymer greatly influence the final strength development. When surface contaminants and hydroxyl group elements, such as pectin, lignin, and hemicellulose, are adequately extracted from the fibers, the interaction between the natural fibers and its polymer matrix improves [21]. Numerous approaches, such as fiber treatment, inclusion of nano-fillers, and hybridization, have been developed to circumvent these restrictions. The utilization of these processes to develop natural fiber composite materials in recent years has expanded their range of applications to include structural, home, aerospace, sports, automotive, and other industries [22,23]. Many chemical modification approaches, such as treating fillers with alkali solution, silane, and acetylation, have been used to improve the filler–polymer interfacial compatibility, which evidently boosts the mechanical and physical characteristics of the bio-composites [24,25]. Moreover, using proper compatibilizers is another way to improve the compatibility and interfacial linkage between bio-fibers and thermoplastics. Among the compatibilizers, a wide range of compounds such as isocyanates and malleated polyolefins have been employed [26,27]. According to previous studies [28,29,30,31], the incorporation of a malleated polypropylene compatibilizer enhanced the load transfer from the matrix to the reinforcing fiber.

Polypropylene grafted with maleic anhydride (MAH) was used as a compatibilizer in bio-composite preparation, in part due to the ability of the carboxylic group (-COOH) of the maleic anhydride on the polymer surface to react with the hydroxyl groups (OH) on the filler surface [32,33]. Additionally, the incorporation of the isocyanate group (-NCO) onto the polymer surface resulted in further improvement in the interfacial bonding between the filler and polymer, as reflected by significant improvements in the mechanical properties [34,35]. Natural fillers behave as nucleate agents for the crystallization of the PP-grafted isocyanate group, enhancing the interfacial adhesion and reducing PP chain mobility. Generally, polymer modification using reagents that contain functional groups is widely used to promote the reaction with the OH groups, which are abundant on the surface of the lignocellulosic material, resulting in chemical bonding between both components [24,36,37].

In this study, new compatibilizers are synthesized by applying grafting techniques on date palm powder (DPP) and the PP polymer matrix. The modified filler and matrix enhance the new bio-based composites by developing better compatibility between both constituents. In particular, and to the best of the authors’ knowledge, this study reports for the first time a functionalization scheme for PP with a 2-isocyanatoethyl methacrylate (IEM) compatibilizer to produce urethane bonding with the natural filler. The grafted bio-composites are tested experimentally to determine the chemical compositions and characterize the morphological, physical, mechanical, and thermal properties to explore the effectiveness of the newly established techniques for chemical bonding between the DPP filler and PP matrix.

## 2. Materials and Methods

### 2.1. Materials

#### 2.1.1. Chemicals and Modifiers

**Scheme 1:**
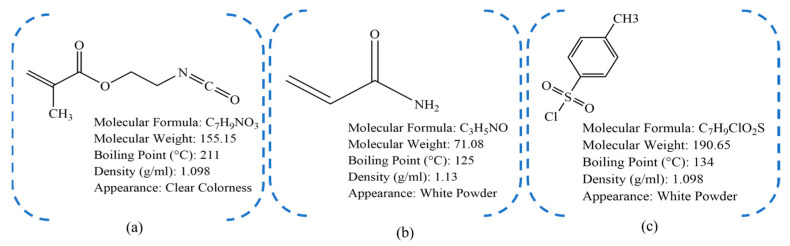
Chemical structure and properties of (**a**) IEM, (**b**) AcAm, and (**c**) TsCl chemical groups.

Several chemicals were used in the current study, including acrylamide (AcAm, 99%, LOBA, Mumbai, India), 2-isocyanatoethyl methacrylate (IEM, 98%, Sigma-Aldrich, USA, St. Louis, MO, USA), and 4-toluenesulfonyl chloride (-TsCl, 99%, Sigma-Aldrich, St. Louis, MO, USA) as functional groups; acetone as a solvent (99%, Sigma-Aldrich, St. Louis, MO, USA); triethylamine (TEA, 99%, Sigma-Aldrich, St. Louis, MO, USA) as a catalyst; and dicumyl peroxide (DCP, 98%, Fisher, Portsmouth, NH, USA) as an initiator. The chemical structure and properties of the used modifiers in this study are depicted in Scheme 1. All chemicals were used as received. The synthesis procedures were performed on a solid-state phase mixture (PP, DPP, and solvent) in a round-bottom flask using magnetic stirring. Good dispersion of the functional groups at the filler–polymer interface is critical to achieve an effective synthesis process [25]. Additionally, while the melt state is commonly used for polymer functionalization, it can cause considerable PP degradation, as well as poor thermomechanical properties. Alternatively, the solid-state phase reaction can be adopted at room temperature to overcome these issues [38].

#### 2.1.2. Filler and Polymer

**Figure 1:**
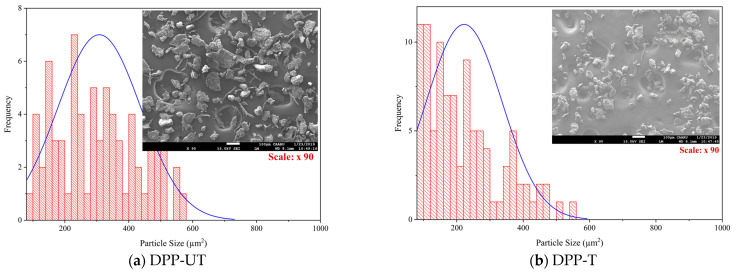
Particle size measurement and distribution: (**a**) DPP-UT; (**b**) DPP-T.

PP homopolymer characterized by a density of 0.905 g/cm^3^ and melt flow rate of 2.15 g/10 min at 230 °C/2.16 kg was supplied by INEOS, London, UK. Natural fibers were mechanically separated from date palm pedicles into tiny pieces and ground using a cryogenic ball milling machine before sifting with a 100-mesh sieve. The produced raw powder was utilized to fill the PP matrix without treatment and was designated as DPP-UT. For another set of samples, the powder was treated following a new chemical treatment process as reported in [39,40] and denoted by DPP-T. Using the Archimedes principle and a hexane solvent, the average densities of untreated and treated date palm powders were measured and reported as 0.6 g/cm^3^ and 0.79 g/cm^3^, respectively. Table 1 displays the chemical compositions of both treated and untreated DPP as determined by an extractive technique described in the literature [41,42]. SEM images of DPP were processed using Mountains^®^ 8 software to estimate the particle size distribution, as shown in Figure 1.

### 2.2. Chemical Functionalization

#### 2.2.1. PP-*g*-IEM

**Scheme 2:**
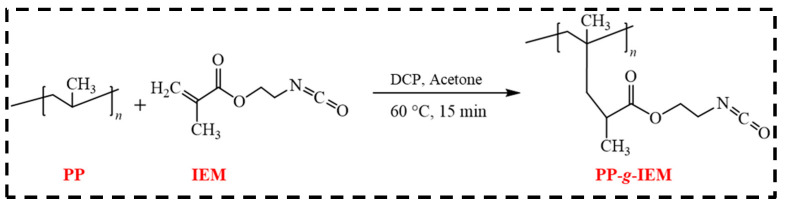
Schematic representation for PP-*g*-IEM.

PP was immersed in acetone to formulate a mixture comprising DCP (0.25 wt.% with reference to PP) as an initiator and IEM (0.5 wt.% with reference to PP) as a functional group. The mixture was stirred in a reflux condenser at 60 °C for 15 min, filtered, and rinsed with distilled water to eliminate any excessive compounds. The conversion reaction procedure is depicted in Scheme 2. It is worthwhile mentioning that the moisture absorbed by the PP-isocyanate group degrades its reactivity, efficiency, and performance. Therefore, mixing DPP and PP should be accomplished immediately after PP functionalization.

#### 2.2.2. PP-*g*-AcAm

**Scheme 3:**
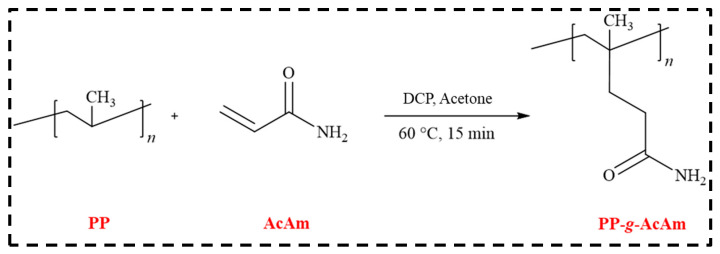
Schematic representation of PP-*g*-AcAm.

In this scheme, synthesis was performed following a process reported in the literature [43], with some modifications in regard to the amount of reactants, reaction temperature, and time. After immersing PP in acetone, DCP (0.25 wt.% with reference to PP) as an initiator and AcAm (3 wt.% with reference to PP) as a functional group were added to the mixture and stirred in a reflux condenser for 15 min at 60 °C. The yield was filtered and cleaned with water in order to remove excess chemicals. Scheme 3 shows a schematic representation of the technique.

#### 2.2.3. (DPP-T)-*g*-TsCl

**Scheme 4:**
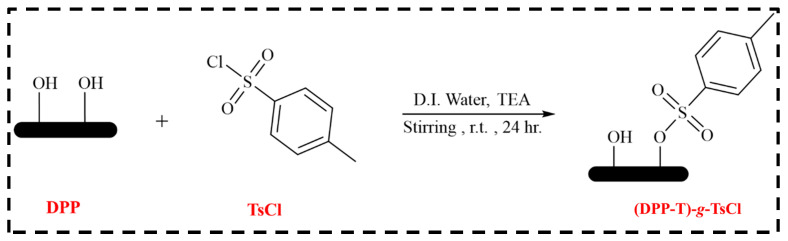
Schematic representation of (DPP-T)-*g*-TsCl.

The synthesis was performed as per procedures reported in [44]. DPP was mixed with TsCl (at 3 times the amount of DPP), trimethylamine (18 mL), and distilled water (200 mL). After 24 h of stirring, the mixture was cleaned and rinsed with hot distilled water followed by warm ethanol to remove the unreacted chemicals. The product was then dried at 105 °C for 24 h. A schematic representation of (DPP-T)-*g*-TsCl is depicted in Scheme 4.

### 2.3. Bio-Composite Fabrication

**Figure 2:**
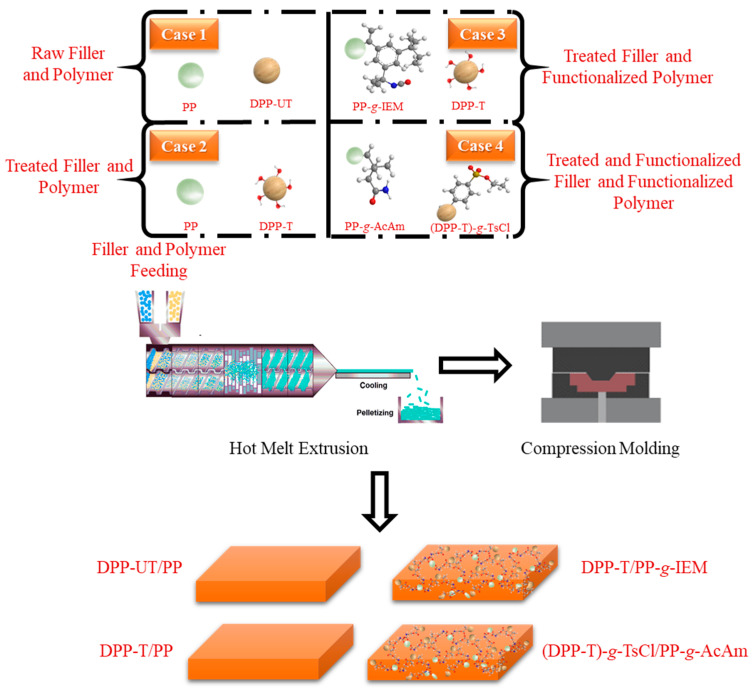
Schematic illustration of the manufacturing process used for the developed bio-composites.

**Scheme 5:**
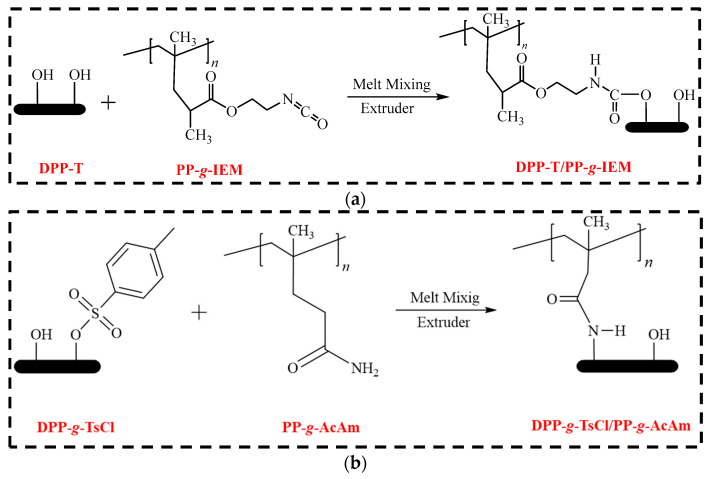
Proposed coupling reactions of (**a**) DPP-T/PP-*g*-IEM and (**b**) (DPP-T)-*g*-TsCl-/PP-*g*-AcAm.

The manufacturing process consisted of hot melt mixing of dried filler and polymer followed by compression molding, forming bio-composite sheets from which test specimens were cut out. The method commenced with the collection of ingredients in designated proportions in order to make a sheet using a mold measuring 270 mm × 270 mm × 3 mm in size. The mixing was performed by employing a laboratory-scale single-screw extruder with two heating zones at 175 °C and 185 °C. The screw’s rotating speed was set to 500 rpm. The extruded material was cooled down to room temperature and manually cut into small granules before compression. The mixture was dried for 15 min at 105 °C in an electric oven to fully remove adsorbed moisture. Lastly, the mold was filled with the melted mixture and pressed in the compression molding machine between two heated plates at 180 °C temperature and 40 MPa pressure. The compression was maintained for 15 min before the heating source was turned off to allow the sheet to cool down. The formed pieces were cured by applying tap water around the exterior surfaces of the heating plates inside the compression molding machine for 2 min. The steps of the fabrication process and the related output are shown in Figure 2. The proposed coupling reactions of DPP-T/PP-*g*-IEM and (DPP-T)-*g*-TsCl/PP-*g*-AcAm are presented in Scheme 5. During melt-phase mixing of PP with treated filler, the hydroxyl group is utilized to react with the -NCO group at the surface of the polymer after grafting with IEM. In the same way, the TsCl group on the DPP surface reacts with the NH_2_ group on the polymer surface. In both cases, crosslinking due to chemical reaction and mechanical interlocking due to fiber treatment will be formed.

### 2.4. Bio-Composite Characterization

FTIR data in the form of an infrared spectrum was used to assess the chemical functional groups attached to the surfaces of the synthesized bio-composites. We instantaneously compiled high spectral resolution data over the full spectrum using a Cary 630 FTIR spectrophotometer.

The instrument was used within a spectral range of 650–4000 cm^−1^ with a resolution of 16 cm^−1^ and 64 scans. The filler–matrix interfacial bonding in the fractured specimens subjected to the tensile test was examined using a scanning electron microscope (SEM) (Model JSM-7800F JEOL, Japan). To avoid electrostatic charge during examination, the broken end of the sample was placed on an aluminum stub and coated with a thin layer of gold.

The mechanical properties were experimentally characterized following the test procedures described in ASTM D638 and ASTM D790 standards, respectively. Five replications were tested for each category of developed bio-composite and the average values of tensile and flexural moduli, tensile and flexural strengths, and elongation at break were determined and reported.

Among the physical properties, the crystallinity degree, density, and water absorption were determined. X-ray diffraction (XRD) was adopted to estimate the crystallinity degree of bio-composite specimens. The XRD spectra were obtained at ambient temperature for the standard sheets (15 mm × 50 mm × 1 mm) using a step detector placed on an X-ray diffractometer (Rigaku Corporation, Japan). The density values of the tested samples were measured using a densitometer. At room temperature, each sample weighing 2 g was dipped in distilled water and the change in volume of the water was measured. The average density values of five replications were calculated and reported. In addition, the water absorption levels of the bio-composite samples were measured in accordance with the ASTM D570 standard. Five dried specimens were placed deep in distilled water at ambient temperature for 24 h. Then, the proportions of water absorbed by the bio-composite specimens were determined by calculating the changes in sample weight before and after immersion in water.

To analyze the thermal decomposition of bio-composites, the thermogravimetric analysis (TGA) was performed to generate analogous derivative thermogravimetric (DTG) plots. The prepared samples were placed in an aluminum container and heated up to 600 °C at a rate of 10 °C/min in a nitrogen-inert environment and then cooled down to ambient temperature.

## 3. Results and Discussion

### 3.1. Chemical Characteristics

**Figure 3:**
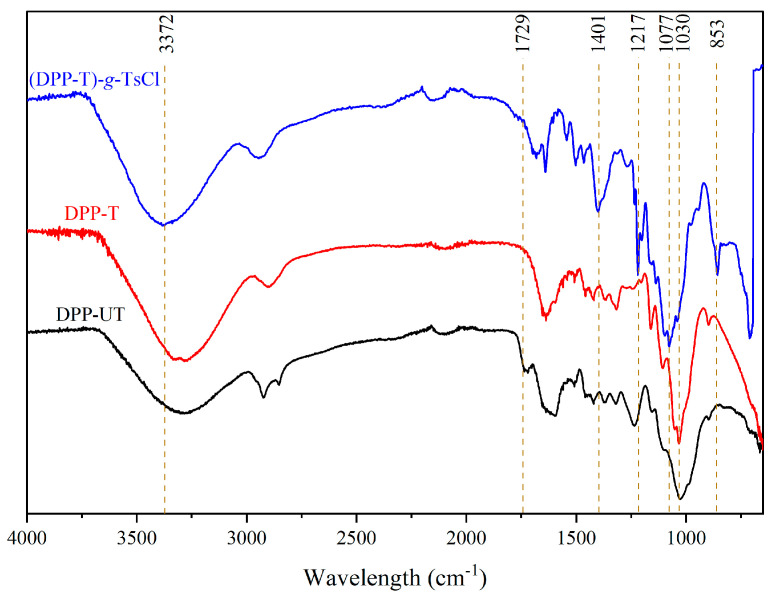
FTIR spectra of DPP-UT, DPP-T, and (DPP-T)-*g*-TsCl.

Figure 3 displays the FTIR spectra of the DPP-UT, DPP-T, and (DPP-T)-*g*-TsCl fibers used in this study. The FTIR spectra of the three samples show the presence of water, as revealed by the peak at 3372 cm^−1^, which is ascribed to the stretching frequency of the hydroxyl group of the natural filler (OH). These bands strongly increased in DPP-T compared to DPP-UT because the treatment eliminated the non-amorphous biomass and increased the filler’s cellulosic content exposure, allowing for easier access to OH groups. The total diminished intensity of around 1729 cm^−1^ for the treated powders was attributed to the elimination of some impurities (dirt). In particular, this peak was designated to C=O stretching of carbonyl and ester bands of the impurities and wax removal due to treatment [40].

The grafting of -TsCl onto the DPP-T surface was successful, as the absorption of SO_2_ bending appeared at 1401 cm^−1^ and 1217 cm^−1^ and the S−O−C stretching at 853 cm^−1^ [45,46]. Finally, all three samples exhibited bands between 1400 and 1500 cm^−1^, indicating C– C stretching of the aromatic ring, with bands at around 1000 cm^−1^ linked to C–H stretching of the cellulose backbone and C–O stretching vibration of cellulose and hemicellulose, respectively [47]. After -TsCl grafting, a slight shift to 1077 cm^−1^ of the cellulose backbone was found, which also confirmed the successful addition of the -TsCl group on the surface of the filler.

**Figure 4:**
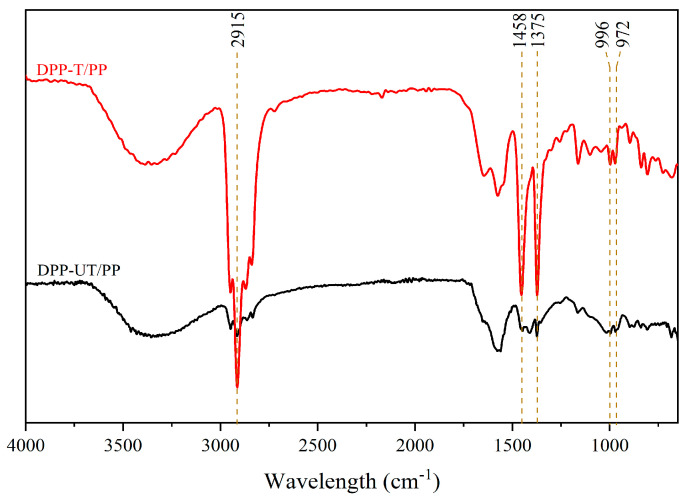
FTIR spectra of DPP-UT/PP and DPP-T/PP.

Figure 4 exhibits the FTIR spectra of the control cases of bio-composites without compatibilizers, indicating similar peak patterns in both cases, although with more evident and stronger peaks for DPP-T/PP. Absorption peaks associated with the presence of the methyl group (CH_3_) in the polypropylene can be observed in both samples. Absorption peaks developed for both bio-composites at 972, 996 and 1375 cm^−1^, which were allotted to rocking and symmetric bending vibration modes of CH_3_ groups. Typically, such absorption bands are indicative of the presence of cellulose, lignin, and hemicellulose. However, the intensity of those peaks became more noticeable in the DPP-T/PP composite compared to DPP-UT/PP due to that treatment that exposed the richness of the cellulose biomass [48]. The sharp peak appearing around 2915 cm^−1^ was attributed to CH_2_ asymmetric stretching of the PP backbone, as reported in the literature. It was noticeably clear that for DPP-T/PP sample this peak was more pronounced compared to the DPP UT/PP sample, which occurred due to the treatment resulting in the removal of most of the impurities. Hence, no overlap of peaks was observed. A similar explanation can be used to justify the noticeable appearance of absorption peaks of CH_2_ deformation stretching in the spectrum range of 1375 to 1458 cm^−1^ for the DPP-T/PP sample [49].

**Figure 5:**
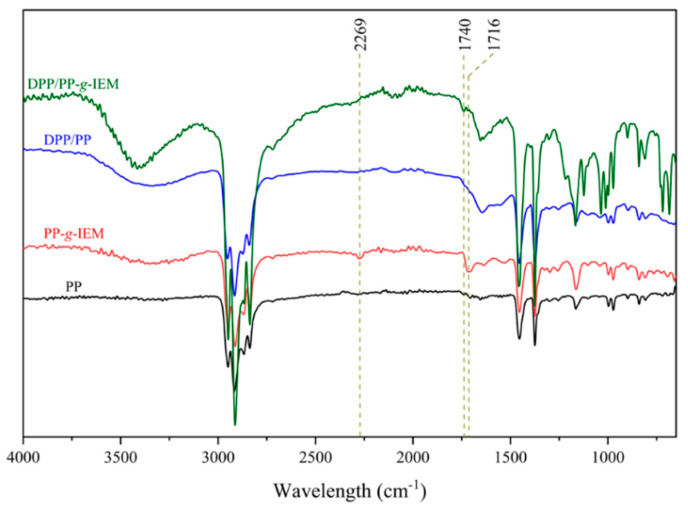
FTIR spectra of PP, PP-*g*-IEM, DPP-T/PP, and DPP-T/PP-*g*-IEM.

Figure 5 depicts the FTIR spectra of PP, PP-*g*-IEM, DPP/PP, and DPP/PP-*g*-IEM. According to the FTIR spectrum of PP-*g*-IEM, N=C=O stretching induced absorption at 2269 cm^−1^, whereas the ester in the C=O triggered absorption at 1716 cm^−1^. This demonstrated that the functionalization was successful and that the N=C=O group was successfully grafted onto PP [27]. The distinctive peak at 2269 cm^−1^ owing to the N=C=O group vanished in the product after melt mixing of DPP and PP-*g*-IEM, with a corresponding significant increase in the C=O peak at 1740 cm^−1^, indicating the production of urethane due to the reaction between N=C=O and OH. Figure 5 also shows the FTIR spectrum of the DPP/PP sample for comparison.

**Figure 6:**
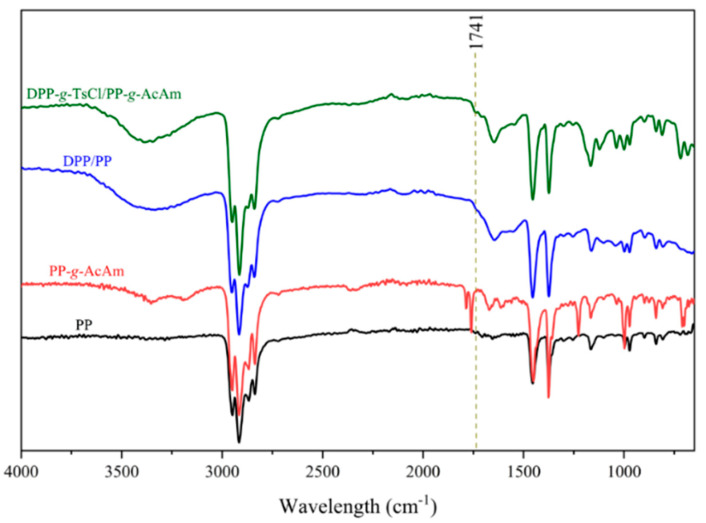
FTIR spectra of PP, PP-*g*-AcAm, DPP/PP, and (DPP-T)-*g*-TsCl/PP-*g*-AcAm.

The FTIR spectrum of the (DPP-T)-*g*-TsCl/PP-*g*-AcAM bio-composite (Figure 6) reveals a peak at 1741 cm^−1^, which can be traced to the stretching frequency of the C=O group from AcAm. This peak validated the formation of amide linkages because of the reaction between -TsCl and -NH_2_ on the filler and polymer surfaces, respectively.

### 3.2. Morphology

#### 3.2.1. Filler Surface

**Figure 7:**
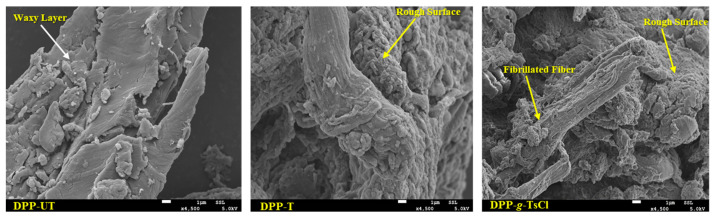
SEM images of the untreated, treated, and functionalized fillers.

The surfaces of the filler were assessed using SEM to explore the morphological changes due to the modification method, as shown in Figure 7. The treated fibers in the figure have a rough appearance where fibrils were exposed at the surface, while the untreated fiber surface is smooth, which indicates that fibrils were covered by a waxy layer. It is essential to remove surface impurities, as these contribute to poor fiber–matrix mechanical interlocking. A higher degree of filler fibrillation can be observed for (DPP-T)-*g*-TsCl because the treatment method improved the filler crystallinity due to partial extraction of hemicellulose and lignin. In addition, both the treatment and functionalization of DPP increased its surface roughness, as depicted in the SEM images.

#### 3.2.2. Fractography

**Figure 8:**
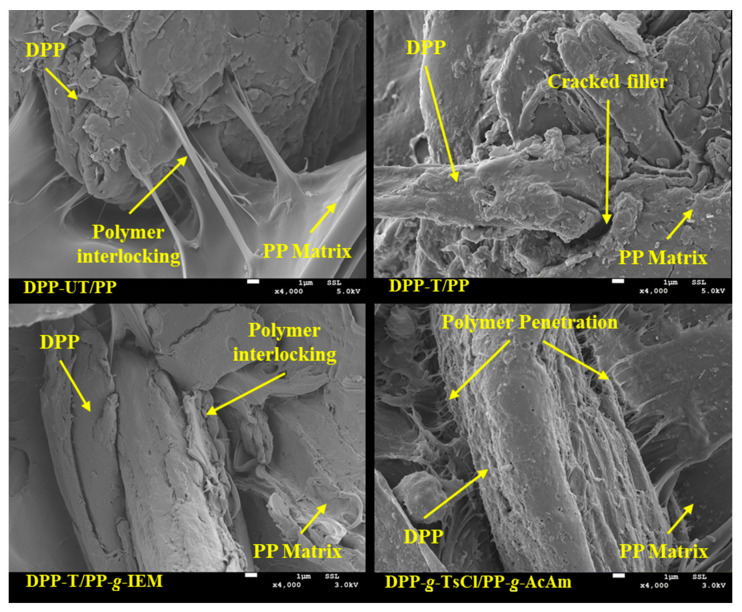
SEM micrographs of fractured surfaces of the tested tensile specimens.

To investigate failure mechanisms in the developed bio-composites under tensile loading, SEM images of fractured surfaces were obtained, as shown in Figure 8. Generally, there is no indication of huge agglomeration of particles on the fracture surfaces in the figure, which indicates homogeneous mixing and good affinity at the particle and matrix interface. This was due to the powder geometry of PP matrix, which facilitated a decent dispersion of the filler, preventing the formation of filler agglomeration. In the micrograph of the untreated bio-composite, fiber pullout due to weak adhesion between the polymer and fiber can be observed. In addition, the removal of impurities and the waxy layer from the particle surface showed the good mechanical interlocking, as indicated by polymer penetration in the filler cell wall.

It can be clearly noticed that the use of compatibilizer enhanced the filler–polymer adhesion by two means: (a) mechanical interlocking, as seen in the SEM images; (b) chemical bonding, as confirmed by FTIR results. However, in the DPP-T/PP-*g*-IEM composites, improved adhesion at the fiber–matrix interface can be observed. For (DPP-T)-*g*-TsCl/PP-*g*-AcAm samples, the interlocking is even more prominent, which was caused by polymer penetration into the filler surface. The morphologies of the fractured composites confirm that both compatibilizers are efficient in improving the bio-composite compatibility.

### 3.3. Mechanical Properties

#### 3.3.1. Tensile Strength

**Figure 9:**
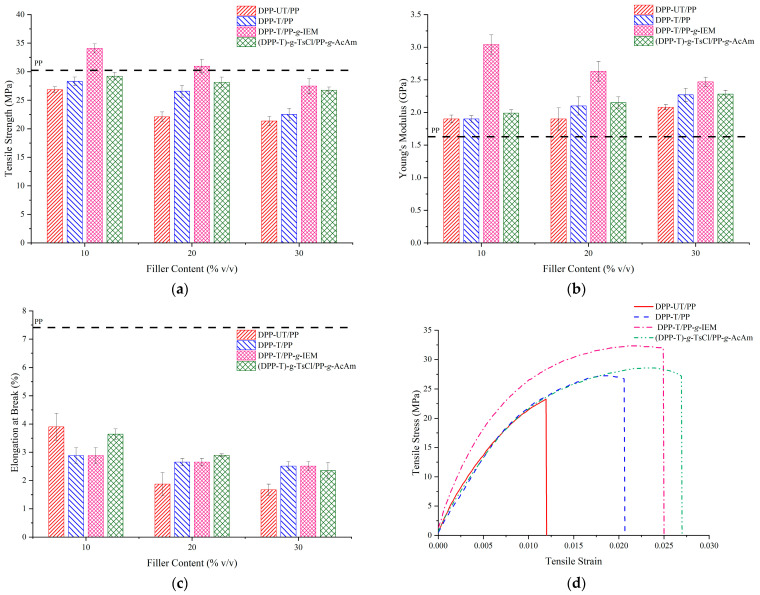
The tensile properties of the developed bio-composites at 20% DPP filler content: (**a**) tensile strength; (**b**) Young’s modulus; (**c**) elongation at break; (**d**) stress–strain curve.

In principle, adhesion at the interface between the fillers and encapsulated polymer as well as the mechanical properties of the constituents can influence the strength and performance of the bio-composites. In particular, the effectiveness of the load transfer from the fillers to the surrounding matrix is determined by the particle size, dispersion or distribution state, surface area, and filler loading. Hence, the use of proper compatibilizers to treat fillers and functionalize both fillers and polymers is intended to develop quality filler–polymer interfaces. The enhanced surface topography of the chemically modified filler results in strong interfacial adhesion and increased tensile strength of the manufactured bio-composites. It is evident that the tensile properties are affected by the filler loading, treatment method, and compatibilizers, as depicted in Figure 9. The tensile strength of the bio-composite reinforced with treated fillers and functionalized PP polymer using isocyanate functional group outperformed other bio-composites in all cases, particularly at 10% DPP loading (Figure 9a). The isocyanate group generates strong bonds between the filler and mating PP matrix with high compatibility, as supported by the SEM micrographs. The strengths of the bio-composite with the filler and polymer coupled by IEM were 2% and 12% greater than those of the neat polymer at fiber loadings of 10% and 20%, respectively. However, further increases in fiber loading led to reductions in strength due to the relatively weak particulate microfibers and their agglomeration in the matrix. Similarly, the use of (DPP-T)-*g*-TsCl and PP-*g*-AcAm increased the tensile strength of the bio-composites in comparison to the untreated and treated filler-based bio-composite, although to a lesser degree compared to DPP-T/PP-*g*-IEM.

The incorporation of stiff particulate fillers in the polymer matrix contributed to the increased tensile modulus of bio-composite, as shown in Figure 9b. The figure demonstrates a similar trend to that for the tensile strength with respect to the effect of treatment and functionalization methods. However, the tensile moduli were improved in the three cases (10, 20, and 30 v.% of filler content), with the highest value found for DPP-T/PP-*g*-IEM relative to the neat polymer. The increases in stiffness of the treated filler-based composites occurred due to the removal of non-cellulosic content found on the surfaces of individual fibers. Additionally, the supplement of compatibilizers influenced interfacial adhesion and subsequently the load transfer and resistance to deformation, as can be clearly noticed from the increased rates of the tensile modulus [50,51]. The elastic modulus is a macro-scale material property, i.e., less responsive to the local stress field, meaning the combination of rigid fillers will make the bio-composite stiffer. Even for the untreated fibers (DPP-UT/PP), an increased elastic modulus was found over the neat PP.

In general, the use of DPP fillers in the PP matrix develops higher tensile properties but reduces the ductility of the bio-composites compared to the neat polymer. As the fiber content increases, the material loses its ductility, as reflected by a decline in the strain at failure (Figure 9c). Removal of the non-cellulosic fibrils makes the fibers more ductile and increases the toughness, as depicted in Figure 9d. However, an increased elongation in (DPP-T)-*g*-TsCl/PP-*g*-AcAm composites was found due to the weakened fiber in contrast to the DPP-T/PP-*g*-IEM counterpart. Clearly, the incorporation of these compatibilizers contributed to the increased toughness of the bio-composite.

#### 3.3.2. Flexural Strength

**Figure 10:**
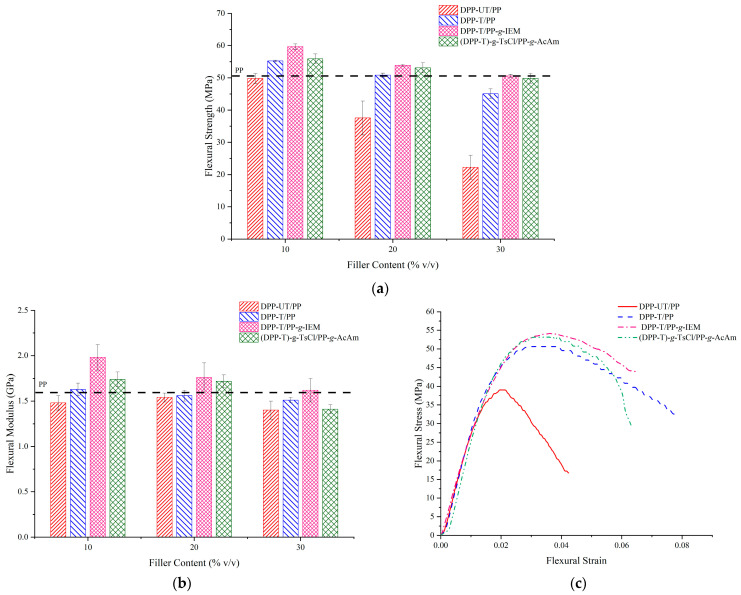
The flexural properties of the developed bio-composites at 20% DPP filler content: (**a**) flexural strength; (**b**) flexural modulus; (**c**) stress–strain curve.

Figure 10a,b exhibits increases in the flexural strength and modulus for the developed bio-composites at 10% and 20% filler contents over the neat polymer, especially in the case of DPP-T/PP-*g*-IEM. These increases in the flexural strength occurred upon functionalization, similar to the observations made for the tensile strength. The flexural strengths decreased with increases in filler content but remained above the threshold strength of the neat polymer. The effects of both fiber modification and polymer functionalization are prominent in enhancing the toughness of the developed bio-composites under bending loading, as shown in Figure 10c. In summary, the new compatibilization methods were able to enhance the filler–polymer adhesion because of the higher tolerated flexural stress levels attained by the bio-composites.

### 3.4. Physical Properties

#### 3.4.1. Density

Density is a critical property of bio-composites that determines their suitability in various industrial applications as replacements for neat polymers or synthetic-filler-reinforced polymers. For all developed bio-composites, decreased density was achieved due to the presence of the natural filler (DPP) as compared to neat PP (Table 2). The untreated fillers with PP showed the lowest density values, followed by the treated filler, as solidification condenses the material. Treated fillers and functionalized polymers caused slight increases in density for the corresponding bio-composites due to the chemical treatment effect, functionalization, and modified bonding mechanism (at 20% and 30% filler loadings). This trend was clearly noticeable for the functionalized filler and functionalized polymer ((DPP-T)-*g*-TsCl/PP-*g*-AcAm). Generally, the developed bio-composites showed close density values in all cases, which are suitable for various products, especially where lightweight structures are sought.

#### 3.4.2. Water Absorption

To expand the range of applications of DPP-based bio-composites, it is essential to limit water intake. The increased water absorption ability of the bio-composites is due to the hydrophilic nature of the fillers and weak interface between the fillers and the matrix. Generally, water absorption increases with increasing filler content [52]. The trends in Table 2 indicate that the (DPP-T)-*g*-TsCl-based functionalized polymer composites exhibited the least water absorption capacity compared to the treated filler and functionalized polymer. Hence, filler modification using either chemical treatment (DPP-T) or functionalization ((DPP-T)-*g*-TsCl) results in greater hydrophobicity for the bio-composites compared to the untreated filler. This serves as another indicator that the surface compatibly was improved upon functionalization with fillers and polymer. The results also show that the water absorption values of DPP-UT/PP composites are slightly higher than the values of the other developed bio-composites. The waxy layer and high content of hemicellulose in the untreated filler, which comprises OH groups, are highly capable of forming hydrogen bonds with water molecules.

#### 3.4.3. XRD

**Figure 11:**
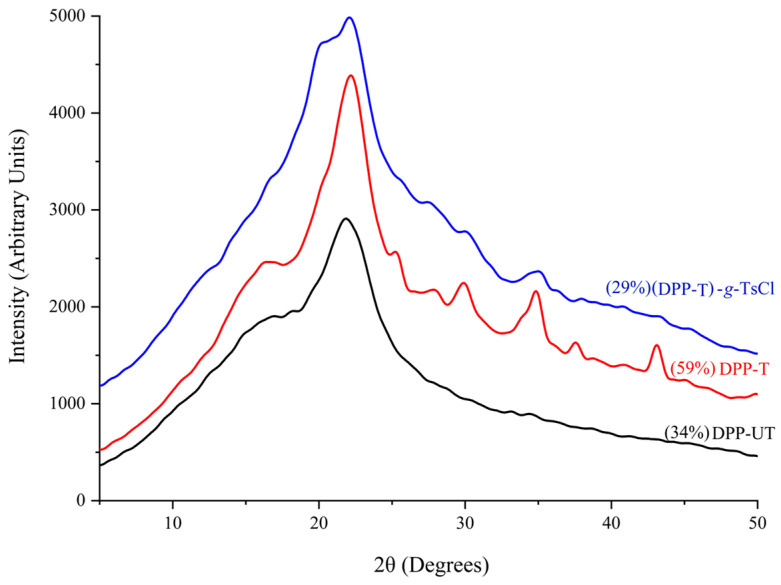
XRD patterns of DPP fillers (the values in brackets indicate the degrees of crystallinity).

XRD was used to evaluate the crystallinity index of the DPP filler and associated bio-composites, which can be linked to the physical and mechanical properties. A higher crystallinity degree suggests a stiffer and more thermally stable but less ductile material, whereas an amorphous biomass provides better impact resistance and elasticity. The X-ray diffraction patterns presented in Figure 11 reflect the impact of the chemical treatment on the crystallinity degree of DPP, which was increased due to the removal of the non-crystalline biomass. However, the TsCl compatibilizer degraded the crystallinity of the filler because of the presence of the chloride group acting as a bleaching agent on the filler, which affected its structural integrity.

Here, (DPP-T)-*g*-TsCl/DPP-*g*-AcAmn showed the lowest crystallinity due to the reduced crystallinity of the (DPP-T)-*g*-TsCl and the functional group (TsCl) effect on the filler, as was observed from the XRD results. Increased crystallinity was observed for DPP-T/PP-*g*-IEM, which might be a sign of crystalline ordering of the atoms due to the treatment of the filler. It is worth mentioning that neither treatment of the DPP nor the functionalization of the PP matrix changed the crystallinity of the developed bio-composites, except for DPP-T/PP-*g*-IEM.

### 3.5. Thermal Degradation

**Figure 12:**
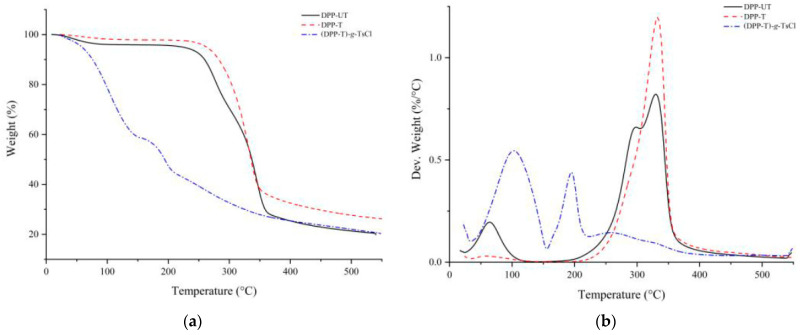
(a) TGA and (b) DTG curves of DPP-UT, DPP-T, and (DPP-T)-*g*-TsCl.

The thermal stability of the used fillers (DPP-UT, DPP-T, and (DPP-T)-*g*-TsCl) was investigated using TGA–DTG (Figure 12). DPP-UT exhibited relatively higher mass loss in the range below of 100 °C owing to the early loss of waxes and pectin, in addition to the vaporization anhydrous water. In addition, two loss stages were distinguished within the range of 200 to 400 °C. The first stage appeared between 200 and 260 °C due to overlapping and delayed decomposition of waxes, pectin, and holocellulose (cellulose and hemicellulose), whereas the second stage, between 260 and 400 °C, was due to the loss of holocellulose and lignin. For DPP-T, a relatively low initial weight loss below 100 °C was found, which occurred due to the disappearance of anhydrous water. Furthermore, a major mass loss was observed between 200 and 400 °C, representing the overlapping and simultaneous decomposition of holocellulose and lignin. The TGA–DTG results for (DPP-T)-*g*-TsCl exhibited a significant mass shortfall of approximately 60% at the temperature range below 200 °C. This fast and drastic degradation was believed to be due to the accelerated breakdown of cellulose, as driven by the chloride molecules acting as bleaching agents [53]. The effect of the chloride molecules on the thermal stability of date palm fiber was observed during processing of the bio-composite within a close temperature range.

**Figure 13:**
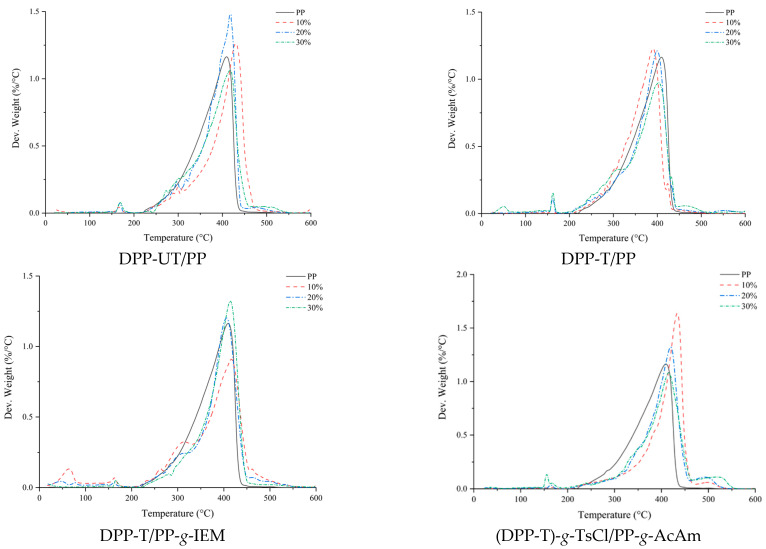
DTG curves of the developed bio-composites.

The DTG results for the developed bio-composites (DPP-UT/PP, DPP-T/PP, DPP-T/PP-*g*-IEM, and (DPP-T)-*g*-TsCl/PP-*g*-AcAm) at different filler loading levels in reference to the neat polymer are illustrated in Figure 13. Slight weight loss below 100 °C was noticed in all bio-composite samples, which occurred due to evaporation of anhydrous water existing as internal moisture in the filler. Another point to note is the small peak around 165 °C, which appeared in all bio-composite samples, representing the melting point of the PP polymer [54]. This peak was not observed in the neat polymer sample, since no mass loss was attributed to this peak and only a phase change occurred due to the melting of the polymer (solid–liquid state). It can be noted that the appearance of this peak for the composite samples was due to the combined overlapping degradation of hemicellulose in the same temperature range assigned to the melting of PP. Above 200 °C, for all composites the degradation occurred in three stages. The first decomposition stage appeared in the range between 225 and 340 °C, which was attributed to the degradation of hemicellulose in the filler. The second stage was between 340 and 450 °C, suggesting simultaneous decomposition of polypropylene, hemicellulose, and cellulose. The third degradation stage occurred above 450 °C, when the polymer and filler were completely decayed with residues of the bio-composites related to the filler ash combined with the decomposition of lignin.

In general, for all bio-composite samples, improved thermal stability was observed, as indicated by a slight shift to the right of the main decomposition peak as the filler content increased. This trend was found in all samples except for the treated filler. As was confirmed earlier, the chemical treatment to applied the fillers eliminated wax, impurities, and pectin, as well as some of the non-cellulosic materials (lignin and hemicellulose). Thus, no major shift or change was observed in the major degradation peak for the treated samples. On the other hand, it was found that the compatibilizers play a crucial role in improving the thermal stability of the developed bio-composites. It is worthwhile noting that functionalizing both the polymer and filler produced composites ((DPP-T)-*g*-TsCl/PP-*g*-AcAm) with improved thermal stability compared to the bio-composites fabricated using only functionalized polymer (DPP-T/PP-*g*-IEM).

## 4. Conclusions

In this research work, two schemes for the surface modification of lignocellulosic DPP and PP were developed and assessed to manufacture bio-composites. The first scheme involved a novel chemical treatment of DPP and functionalization of PP using IEM compatibilizer. This scheme had never been performed before, and this is the first report on the use of this new compatibilizer. The first process resulted in good performance for the developed bio-composites, as the mechanical properties, including the tensile strength and modulus and flexural strength and modulus, exceeded the thresholds of neat PP. In the second scheme, the functionalization of both DPP-T and PP resulted in higher water absorption resistance but lower tensile strength compared to the first scheme. In both schemes, FTIR spectra confirmed the occurrence of the chemical bonds and the filler treatment effect on the surface. Furthermore, an XRD test was performed to elucidate the physical behavior of chemically treated DPP. The results showed that the crystallinity was altered due to the introduced chemical crosslinking. The DPP-T/PP-*g*-IEM crystallinity increased the most, which agreed well with the obtained mechanical properties. The SEM results confirmed the filler–polymer compatibility of both schemes. The rough surfaces of both DPP-T and (DPP-T)-*g*-TsCl promote mechanical interlocking, which is crucial in enhancing the performance of the developed bio-composites. The DTG analysis showed slight improvements in thermal stability for the developed bio-composites because of the chemical linkage. Furthermore, the chemical modification of the fibers affected the polarity by exposing OH functional groups and introducing chemical crosslinking at the filler–polymer interface. However, the objective of functional groups is to improve filler–polymer wettability, whereas compatibilizers are often employed to improve the mixing efficiency and bio-composite manufacturing performance, irrespective of the surface compatibility.

Further research works can be pursued to investigate the application of such functionalization techniques to other types of polymers and natural fillers to provide a better bio-composites. Optimal compatibilizers should be produced, evaluated, and properly integrated into hybrid bio- and nano-composites.

## Figures and Tables

**Table 1:** Chemical compositions (%) of untreated and treated date palm powders (NA: not applicable).

Fillers	DPP-UT	DPP-T
Particle Size (µm^2^)	303.8 ± 120.0	221.9 ± 104.7
Hemicellulose	16.57 ± 0.4	10.77 ± 0.4
Lignin	25.02 ± 0.6	24.34 ± 1.4
Cellulose	45.79 ± 0.9	64.89 ± 1.3
Pectin	4.95 ± 0.69	NA
Wax	5.40 ± 0.28	NA
Water-Soluble Matter	2.27 ± 0.02	NA

**Table 2:** Summary of the physical properties of the developed bio-composites.

	Filler Content (%)	Density (g/cm^3^)	Water Absorption (%)	Crystallinity Degree (%)
PP	-	0.902	0.016	44.8
DPP-UT/PP	10	0.875	0.352	43.2
	20	0.877	0.577	41.0
	30	0.859	0.683	41.5
DPP-T/PP	10	0.883	0.286	44.5
	20	0.879	0.459	42.5
	30	0.876	0.664	41.2
DPP-T/PP-*g*-IEM	10	0.905	0.293	45.4
	20	0.895	0.447	46.4
	30	0.883	0.595	54.2
(DPP-T)-*g*-TsCl/PP-*g*-AcAm	10	0.894	0.247	33.7
	20	0.88	0.354	36.1
	30	0.881	0.413	41.4

## Data Availability

Not applicable.

## References

[1] Khalid M.Y., Al Rashid A., Arif Z.U., Ahmed W., Arshad H., Zaidi A.A. (2021). Natural Fiber Reinforced Composites: Sustainable Materials for Emerging Applications. Results Eng..

[2] Sadik T., Muthuraman S., Sivaraj M., Negash K., Balamurugan R., Bakthavatsalam S. (2022). Mechanical Behavior of Polymer Composites Reinforced with Coir and Date Palm Frond Fibers. Adv. Mater. Sci. Eng..

[3] Barton-Pudlik J., Czaja K. (2018). Fast-Growing Willow (Salix Viminalis) As A Filler in Polyethylene Composites. Compos. Part B Eng..

[4] Saba N., Jawaid M. (2018). A Review on Thermomechanical Properties of Polymers and Fibers Reinforced Polymer Composites. J. Ind. Eng. Chem..

[5] Alzebdeh K.I., Nassar M.M.A., Al-Hadhrami M.A., Al-Aamri O., Al-Defaai S., Al-Shuaily S. (2017). Characterization of Mechanical Properties of Aligned Date Palm Frond Fiber-Reinforced Low-Density Polyethylene. J. Eng. Res..

[6] Ashori A., Nourbakhsh A. (2010). Reinforced Polypropylene Composites: Effects of Chemical Compositions and Particle Size. Bioresour. Technol..

[7] Nassar M.M.A., Alzebdeh K.I., Pervez T., Al-Hinai N., Munam A. (2021). Progress and Challenges in Sustainability, Compatibility, and Production of Eco-Composites: A State-Of-Art Review. J. Appl. Polym. Sci..

[8] Fiore V., Badagliacco D., Sanfilippo C., Miranda R., Valenza A. (2021). An Innovative Treatment Based on Sodium Citrate for Improving the Mechanical Performances of Flax Fiber Reinforced Composites. Polymers.

[9] Keskisaari A., Kärki T. (2018). The Use of Waste Materials in Wood-Plastic Composites and their Impact on the Profitability of the Product. Resour. Conserv. Recycl..

[10] Yang G., Park M., Park S.J. (2019). Recent Progresses of Fabrication and Characterization of Fibers-Reinforced Composites: A Review. Compos. Commun..

[11] Lassoued M., Mnasri T., Hidouri A., Ben Younes R. (2018). Thermomechanical Behavior of Tunisian Palm Fibers Before and After Alkalization. Constr. Build. Mater..

[12] Zegaoui A., Derradji M., Ma R., Cai W.A., Liu W.-B., Wang J., Dayo A.Q., Song S., Zhang L.L. (2018). High-Performance Polymeric Materials with Greatly Improved Mechanical and Thermal Properties from Cyanate Ester/Benzoxazine Resin Reinforced by Silane-Treated Basalt Fibers. J. Appl. Polym. Sci..

[13] Balla V.K., Kate K.H., Satyavolu J., Singh P., Tadimeti J.G.D. (2019). Additive Manufacturing of Natural Fiber Reinforced Polymer Composites: Processing and Prospects. Compos. Part B Eng..

[14] Khieng T.K., Debnath S., Ting Chaw Liang E., Anwar M., Pramanik A., Basak A.K. (2021). A Review on Mechanical Properties of Natural Fibre Reinforced Polymer Composites Under Various Strain Rates. J. Compos. Sci..

[15] Carbonell-Verdú A., García-García D., Jordá A., Samper M.D., Balart R. (2015). Development of Slate Fiber Reinforced High Density Polyethylene Composites for Injection Molding. Compos. Part B Eng..

[16] Alzebdeh K.I., Nassar M.M.A. (2021). Polymer Blend Natural Fiber Based Composites. Fiber Reinforced Composites Constituents, Compatibility, Perspectives, and Applications.

[17] Liao J., Brosse N., Pizzi A., Hoppe S., Xi X., Zhou X. (2019). Polypropylene Blend with Polyphenols through Dynamic Vulcanization: Mechanical, Rheological, Crystalline, Thermal, and UV Protective Property. Polymers.

[18] Sider I., Nassar M.M.A. (2021). Chemical Treatment of Bio-Derived Industrial Waste Filled Recycled Low-Density Polyethylene: A Comparative Evaluation. Polymers.

[19] Zhang H. (2014). Effect of A Novel Coupling Agent, Alkyl Ketene Dimer, on the Mechanical Properties of Wood-Plastic Composites. Mater. Des..

[20] Panaitescu D.M., Nicolae C.A., Vuluga Z., Vitelaru C., Sanporean C.G., Zaharia C., Florea D., Vasilievici G. (2016). Influence of Hemp Fibers with Modified Surface on Polypropylene Composites. J. Ind. Eng. Chem..

[21] Nurazzi N.M., Asyraf M.R.M., Rayung M., Norrrahim M.N.F., Shazleen S.S., Rani M.S.A., Shafi A.R., Aisyah H.A., Radzi M.H.M., Sabaruddin F.A. (2021). Thermogravimetric Analysis Properties of Cellulosic Natural Fiber Polymer Composites: A Review on Influence of Chemical Treatments. Polymers.

[22] Mulenga T.K., Ude A.U., Vivekanandhan C. (2021). Techniques for Modelling and Optimizing the Mechanical Properties of Natural Fiber Composites: A Review. Fibers.

[23] Li M., Pu Y., Thomas V.M., Yoo C.G., Ozcan S., Deng Y., Nelson K., Ragauskas A.J. (2020). Recent Advancements of Plant-Based Natural Fiber–Reinforced Composites and their Applications. Compos. Part B Eng..

[24] Guo C.G., Wang Q.W. (2008). Influence of m-Isopropenyl-α, α-Dimethylbenzyl Isocyanate Grafted Polypropylene on The Interfacial Interaction of Wood-Flour/Polypropylene Composites. J. Appl. Polym. Sci..

[25] Oladele I.O., Ibrahim I.O., Akinwekomi A.D., Talabi S.I. (2019). Effect of Mercerization on the Mechanical and Thermal Response of Hybrid Bagasse Fiber/CaCO_3_ Reinforced Polypropylene Composites. Polym. Test..

[26] Aggarwal P.K., Raghu N., Karmarkar A., Chuahan S. (2013). Jute-Polypropylene Composites Using m-TMI-Grafted-Polypropylene as A Coupling Agent. Mater. Des..

[27] Sohail M., Ashfaq B., Azeem I., Faisal A., Doğan S.Y., Wang J., Duran H., Yameen B. (2019). A Facile and Versatile Route to Functional Poly(Propylene) Surfaces Via UV-Curable Coatings. React. Funct. Polym..

[28] Gironès J., Lopez J.P., Vilaseca F., Bayer R., Herrera-Franco P.J., Mutjé P. (2011). Biocomposites from Musa Textilis and Polypropylene: Evaluation of Flexural Properties and Impact Strength. Compos. Sci. Technol..

[29] Kaewkuk S., Sutapun W., Jarukumjorn K. (2013). Effects of Interfacial Modification and Fiber Content on Physical Properties of Sisal Fiber/Polypropylene Composites. Compos. Part B Eng..

[30] Lee H.J., Lee H.K., Lim E., Song Y.S. (2015). Synergistic Effect of Lignin/Polypropylene as A Compatibilizer in Multiphase Eco-Composites. Compos. Sci. Technol..

[31] Dayo A.Q., Zegaoui A., Nizamani A.A., Kiran S., Wang J., Derradji M., Cai W.-A., bin Liu W. (2018). The Influence of Different Chemical Treatments on the Hemp Fiber/Polybenzoxazine Based Green Composites: Mechanical, Thermal and Water Absorption Properties. Mater. Chem. Phys..

[32] Mulinari D.R., Cipriano J.D.P., Capri M.R., Brandão A.T. (2017). Influence of Surgarcane Bagasse Fibers with Modified Surface on Polypropylene Composites Influence of Surgarcane Bagasse Fibers with Modified Surface on Polypropylene Composites. J. Nat. Fibers.

[33] El-Sabbagh A. (2014). Effect of Coupling Agent on Natural Fibre in Natural Fibre/Polypropylene Composites on Mechanical and Thermal Behavior. Compos. Part B Eng..

[34] Karmarkar A., Chauhan S.S., Modak J.M., Chanda M. (2007). Mechanical Properties of Wood-Fiber Reinforced Polypropylene Composites: Effect of A Novel Compatibilizer with Isocyanate Functional Group. Compos. Part A.

[35] Liu W., Chen T., Wen X., Qiu R., Zhang X. (2014). Enhanced Mechanical Properties and Water Resistance of Bamboo Fiber–Unsaturated Polyester Composites Coupled by Isocyanatoethyl Methacrylate. Wood Sci. Technol..

[36] Chauhan S., Aggarwal P., Karmarkar A. (2016). The Effectiveness of m-TMI-Grafted-PP as a Coupling Agent for Wood Polymer Composites. J. Compos. Mater..

[37] Nassar M.M.A., Munam A., Alzebdeh K.I., Al Fahdi R., Abu Tarboush B.J. (2021). Efficient Methods of Surface Functionalization of Lignocellulosic Waste Toward Surface Clickability Enhancement. Compos. Interfaces.

[38] Sun M., Zhang X., Chen W., Feng L. (2015). Surface Functionalization of Polypropylene-Bearing Isocyanate Groups in Solid State and their Cyclotrimerization with Diisocyanates. J. Appl. Polym. Sci..

[39] Nassar M.M.A., Alzebdeh K.I., Al-Hinai N., Pervez T. (2021). A New Treatment Method for Cellulose-Rich Fibers Toward High Performance Bio-composite Applications. PPU Res. Repos..

[40] Nassar M.M.A., Alzebdeh K., Munam A., Al-Hinai N., Pervez T., Al-Jahwari F. (2020). Preparation of High-Performance Fiber from Natural Fiber (Date Palm). World Intellectual Property Organization (WIPO).

[41] Jiang W., Han G., Zhang Y., Wang M. (2010). Fast Compositional Analysis of Ramie Using Near-Infrared Spectroscopy. Carbohydr. Polym..

[42] Wang H., Yao X., Sui G., Yin L., Wang L. (2015). Properties of Xanthoceras Sorbifolia Husk Fibers with Chemical Treatment for Applications in Polymer Composites. J. Mater. Sci. Technol..

[43] Yin Z., Zhang X., Yin J., Zhang Y. (1996). Synthesis and Characterization of Polypropylene Grafted by Acrylamide. Polym. Plast. Technol. Eng..

[44] Rahman N.S.A., Azahari B., Yhaya M.F., Ismail W.R. (2016). Crosslinking of Kapok Cellulose Fiber Via Azide Alkyne Click Chemistry as A New Material for Filtering System: A Preliminary Study. Int. J. Adv. Sci. Eng. Inf. Technol..

[45] Gericke M., Schaller J., Liebert T., Fardim P., Meister F., Heinze T. (2012). Studies on the Tosylation of Cellulose in Mixtures of Ionic Liquids and A Co-Solvent. Carbohydr. Polym..

[46] Elchinger P.H., Faugeras P.A., Zerrouki C., Montplaisir D., Zerrouki R., Brouillette F., Zerrouki R. (2012). Tosylcellulose Synthesis in Aqueous Medium. Green Chem..

[47] Krylova V., Dukštienė N. (2013). Synthesis and Characterization of Ag2S Layers Formed on Polypropylene. J. Chem..

[48] Gopanna A., Mandapati R.N., Thomas S.P., Rajan K., Chavali M. (2019). Fourier Transform Infrared Spectroscopy (FTIR), Raman Spectroscopy and Wide-Angle X-Ray Scattering (WAXS) of Polypropylene (PP)/Cyclic Olefin Copolymer (COC) Blends for Qualitative and Quantitative Analysis. Polym. Bull..

[49] Shi J., Xing D., Li J. (2012). FTIR Studies of the Changes in Wood Chemistry from Wood Forming Tissue under Inclined Treatment. Energy Procedia.

[50] Yeo J., Seong D., Hwang S. (2015). Chemical Surface Modification of Lignin Particle and Its Application as Filler in the Polypropylene Composites. J. Ind. Eng. Chem..

[51] Tran L.Q.N., Fuentes C.A., Dupont-gillain C., van Vuure A.W., Verpoest I. (2013). Understanding the Interfacial Compatibility and Adhesion of Natural Coir Fibre Thermoplastic Composites. Compos. Sci. Technol..

[52] Mustafa Z., Taufiq M.J., Mansor M.R. (2018). Influence of Water Absorptivity on Kenaf Fibre Reinforced Recycled-Polymer Composite Properties. Prog. Ind. Ecol. Int. J..

[53] Amarasekara A.S., Ebede C.C. (2009). Bioresource Technology Zinc Chloride Mediated Degradation of Cellulose At 200 °C and Identification of the Products. Bioresour. Technol..

[54] Sun G., Kong X., Wang Z., Luo Q., Li Q. (2021). Experimental Investigation into Stamping of Woven CF/PP Laminates: Influences of Molding Temperature on Thermal, Mesoscopic and Macroscopic Properties. Compos. Struct..

